# Case report: Functional characterization of a *de novo* c.145G>A p.Val49Met pathogenic variant in a case of PIGA-CDG with megacolon

**DOI:** 10.3389/fgene.2022.971473

**Published:** 2022-10-17

**Authors:** Roberta Salinas-Marín, Yoshiko Murakami, Carlos Alberto González-Domínguez, Mario Ernesto Cruz-Muñoz, Héctor Manuel Mora-Montes, Eva Morava, Taroh Kinoshita, Susana Monroy-Santoyo, Iván Martínez-Duncker

**Affiliations:** ^1^ Laboratorio de Glicobiología Humana y Diagnóstico Molecular, Centro de Investigación en Dinámica Celular, Instituto de Investigación en Ciencias Básicas y Aplicadas, Universidad Autónoma del Estado de Morelos, Cuernavaca, México; ^2^ Research Institute for Microbial Diseases, Osaka University, Osaka, Japan; ^3^ Instituto de Biotecnología, Universidad Nacional Autónoma de México, Cuernavaca, México; ^4^ Facultad de Medicina, Universidad Autónoma del Estado de Morelos, Cuernavaca, México; ^5^ Departamento de Biología, División de Ciencias Naturales y Exactas, Campus Guanajuato, Universidad de Guanajuato, Guanajuato, México; ^6^ Department of Clinical Genomics, Mayo Clinic, Rochester, MN, United States; ^7^ Department of Medical Genetics, University of Pecs Medical School, Pecs, Hungary; ^8^ Frontiers in Congenital Disorders of Glycosylation Consortium, National Institute of Neurological Diseases and Stroke (NINDS), National Institute of Child Health and Human Development (NICHD) and the National Center for Advancing Translational Sciences (NCATS), and the Rare Disorders Clinical Research Network (RDCRN), Bethesda, MD, United States; ^9^ Centro de Investigación Traslacional, Instituto Nacional de Pediatría, Secretaría de Salud, Mexico City, México

**Keywords:** GPI (glycosylphosphatidylinositol), CDG (congenital disorder of glycosylation), exome, PIGA, CD59 antigen

## Abstract

A subgroup of congenital disorders of glycosylation (CDGs) includes inherited GPI-anchor deficiencies (IGDs) that affect the biosynthesis of glycosylphosphatidylinositol (GPI) anchors, including the first reaction catalyzed by the X-linked *PIGA*. Here, we show the first PIGA-CDG case reported in Mexico in a male child with a moderate-to-severe phenotype characterized by neurological and gastrointestinal symptoms, including megacolon. Exome sequencing identified the hemizygous variant *PIGA* c.145G>A (p.Val49Met), confirmed by Sanger sequencing and characterized as *de novo*. The pathogenicity of this variant was characterized by flow cytometry and complementation assays in PIGA knockout (KO) cells.

## Introduction

The glycosylphosphatidylinositol (GPI) structure is ubiquitous among eukaryotes with a common minimal backbone consisting of three mannoses, one non-*N*-acetylated glucosamine (GlcN), and inositol phospholipid (PI). GPIs are attached to proteins *via* an amide bond between the C-terminal carboxyl group and an amino group of ethanolamine phosphate, and their fatty chains of PI are inserted into the outer leaflet of the plasma membrane. In this way, more than 150 different human proteins with diverse functions are anchored through GPIs (Stevens, 1995; Fujita and Kinoshita, 2012; Kinoshita and Fujita, 2016). The biosynthesis of GPIs is a stepwise sequence of 11 reactions (Kinoshita, 2020). The first reaction consists in the transference of *N*-acetylglucosamine (GlcNAc) from UDP-*N*-acetylglucosamine (UDP-GlcNAc) to the 6-position of inositol to generate GlcNAc-PI and is catalyzed by GPI *N*-acetylglucosaminyl transferase (GPI-GnT), a complex monoglycosyltransferase, consisting of seven subunits, of which the X-linked PIGA is a catalytic subunit (Miyata et al., 1993).

Somatic mutations in *PIGA* can occur, leading to paroxysmal nocturnal hemoglobinuria, an acquired clonal disease of hematopoietic stem cells (Takeda et al., 1993; Hill et al., 2017). Additionally, *PIGA* pathogenic germline variants have been reported in humans and are part of a subgroup of inherited GPI-anchor deficiencies (IGDs) classified as congenital disorders of glycosylation (CDGs) (Freeze et al., 2014; Tarailo-Graovac et al., 2015; Ng and Freeze, 2018). Twenty-one out of 27 genes involved in GPI biosynthesis have been reported with pathogenic germline variants, with PIGA-CDG being the only X-linked IGD (Kinoshita, 2020). In PIGA-CDG, only males have been found to be clinically affected (Tarailo-Graovac et al., 2015).

The phenotypical spectrum in PIGA-CDG ranges from a mild-to-moderate developmental delay (DD), treatable epilepsy, with no dysmorphic features, and no organ malformations in the milder end of the spectrum to profound DD/intellectual disability, treatment-refractory epilepsy, dysmorphic features, and multi-organ malformations in the most severe end of the spectrum (Bayat et al., 2020). We report, herein, the first Mexican child with PIGA-CDG presenting a previously uncharacterized novel missense *PIGA* pathogenic variant, resulting in a moderate-to-severe phenotype.

## Materials and methods

### Sequencing

Genomic DNA (gDNA) was extracted from the patient’s saliva and enriched for targeted regions using a hybridization-based *in house* Invitae® protocol for clinical exome sequencing (CES) analysis and sequenced (NextSeq Instrument, Illumina, San Francisco, CA, United States). Confirmation of the variant in the patient and parental screening was performed by the gDNA-based polymerase chain reaction (PCR) product covering exon 2 of *PIGA* obtained using forward primer 5′-GAG​GAG​GAG​CTG​GGA​ATG​G -3′ and reverse primer *PIGA* as 5′-CTG​GTT​GTA​CAT​GAC​TTT​CAG​AG-3′. The 290-bp amplicon was isolated and sequenced using an ABI Prism 3130xl autoanalyzer (Applied Biosystems, Foster City, CA, United States), and the results were visualized using SnapGene Viewer 2.2.2 (GSL Biotech LLC, Chicago, IL, United States).

### Glycophosphatidylinositol anchored protein expression and rescue analysis

The CD16 expression in granulocytes was performed on one blood sample per individual. Granulocytes were stained with 0.2 μg of phycoerythrin-anti-CD16 (CD16-PE; clone DJ130c, sc20052, Santa Cruz, United States) for 30 min at 4°C, washed three times with PBS buffer supplemented with 0.5% BSA, and stored in 2% of paraformaldehyde. The cells (20,000 events per sample) were analyzed, and fluorescence data were recorded as individual cellular events on a BD Accuri C6 Plus flow cytometer (Becton Dickinson, Franklin Lakes, NJ, United States). Data were analyzed with FlowJo software according to Neuhofer et al., (2020).

The HEK-293 PIGA knockout (KO) model was generated by the CRISPR/Cas9 system. HEK293 PIGA KO cells were transfected with wild and mutant PIGA cDNA driven by the weak TATA box promoter (pTA). Two days later, the surface expression of GPI-anchored proteins (GPI-APs) was determined by staining cells with mouse anti-CD59 (5H8) and anti-DAF (IA10), followed by a PE-conjugated anti-mouse IgG antibody (BD Biosciences). The cells were analyzed by a flow cytometer (MACSQuant Analyzer; Miltenyi Biotec) with Flowjo software (BD Life Sciences). Lysates from transfectants of wild and mutant pMEHA-PIGA were applied to SDS PAGE, and Western blotting was performed. *PIGA* proteins were detected by rabbit anti-HA polyclonal antibody (MBL), followed by HRP-conjugated anti-rabbit IgG. For the loading control, GAPDH was detected by mouse anti-GAPDH (AM4300, Invitrogen), followed by HRP-conjugated anti-mouse IgG.

## Case description

The patient is the second born of a healthy, young, non-consanguineous couple; family history was unremarkable. Pregnancy and vaginal delivery at term were uneventful; birth weight was 3,500 g (Z-score = 0), height 51 cm (z-score = 0), and Apgar 9/9. At delivery, a thick and copious layer of vernix caseosa covered him. He was admitted to the NICU during his first week of life due to indirect hyperbilirubinemia (indirect bilirubin <20 mg/dl but >14 mg/dl), requiring three cycles of phototherapy and treatment with phenobarbital due to Crigler–Najjar syndrome suspicion. Finally, jaundice resolved when he was 3 months old, and molecular diagnosis of Gilbert’s syndrome (MIM [143500]) was confirmed by identification of the TA6/TA7 genotype in the UDP glucuronosyltransferase family 1 member A1 gene (*UGT1A1*); phenobarbital was stopped, triggering seizures 4 weeks later. A cerebral MRI and two EEGs, performed at 4 and 9 months of age, respectively, revealed a normal myelination process related to the patient’s age and normal cerebral electrical activity.

From 7 months of age, axial hypotonia and limb hypertonia, indifference to the environment, and severe constipation were evident; he was unable to roll over, sit independently, grab objects, and carry them to his middle line, and he only babbled occasionally. Height 73.5 cm (z-score = 2), weight 9,620 g (z-score = 2), and head circumference 45.4 cm (z-score = 1), with a broad and bulging forehead, arched eyebrows, sunken eyes, rough facies, thick and fleshy ears with “elfin” upper tip, wide mouth with thin upper vermillion, short neck, wide thorax without inverted nipples, deep palmar creases, deep-set toe nails, and skin and adipose tissue “doughy” to the touch.

At 15 months of age, a neuropsychological evaluation was executed, using the Battelle Developmental Inventory, 2nd Edition (BDI-2), concluding a significant delay in global developmental quotient (<0.1 percentile) in relation to the patient’s chronological age.

Normal audition and vision were assessed by evoked brainstem potentials. No congenital heart disease or myocardiopathy were documented. Bilateral ureterocele, predominantly right, left ureteropielectasis, and changes in the left ureteral caliber, suggesting vesicoureteral reflux, were identified. No other malformations were reported.

Partial seizures characterized by gaze deviation to the left and motor orofacial automatisms as well as secondary generalized myoclonic seizures presented at the time of diagnosis. The EEG revealed generalized cerebral dysfunction, characterized by epileptiform paroxysms illustrated by periodic lateralized wave-spike discharges in the right and left temporal and parietal regions that tended to generalize. Treatment with levetiracetam was initiated, and later valproate was added due to partial pharmacological response; finally, clonazepam was warranted as rescue treatment in case of sudden, uncontrolled seizures. Good seizure control was achieved, but fever sensitivity and febrile-induced seizures were observed upon upper respiratory tract viral infections.

**FIGURE 1:**
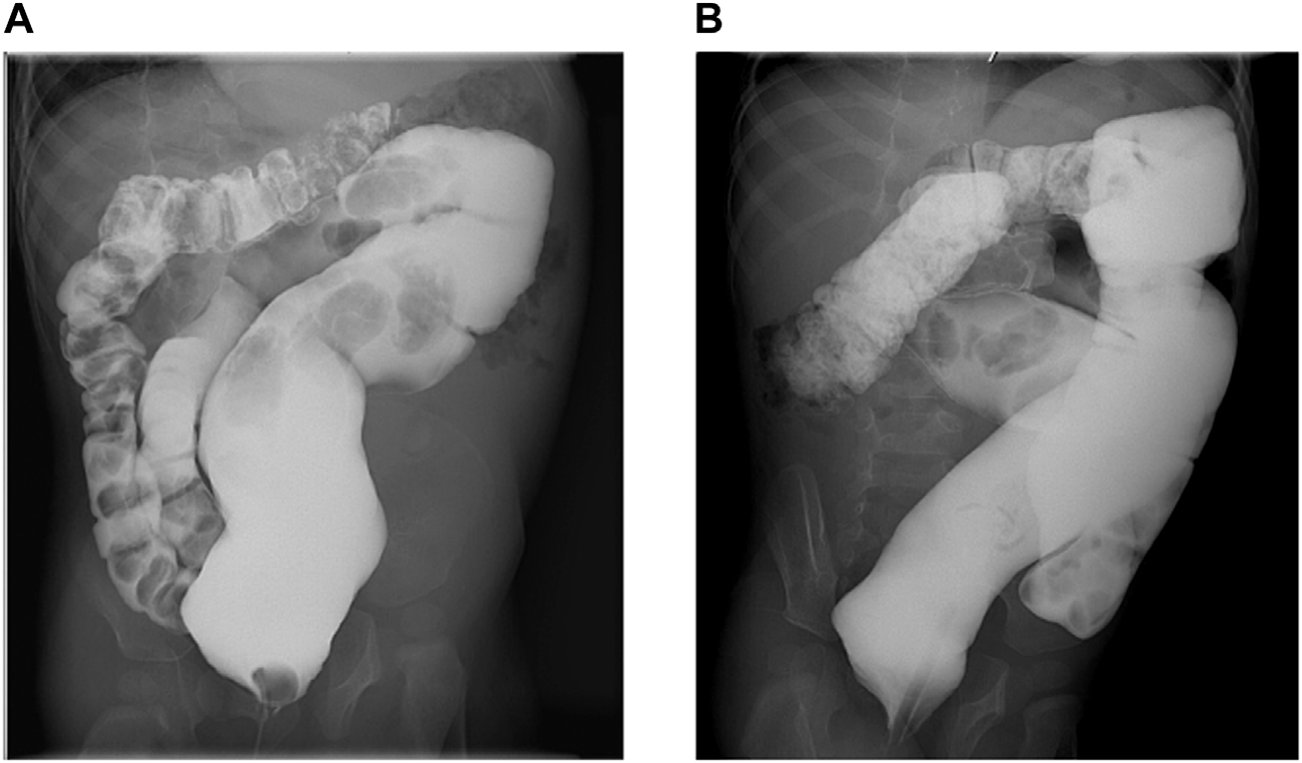
Barium enema. **(A)**. Anteroposterior view showing chronic megarectum. **(B)**. Oblique view showing severe dilatation of the sigmoid and rectum with loss of haustral markings.

Currently, at 34 months of age, he is under neurologic and metabolic surveillance and enrolled in a physical therapy program. He still suffers from severe chronic constipation that has caused a megacolon (Figures 1A,B), requiring treatment with polyethylene glycol 3350 and/or sennosides and glycerine enemas. Other invasive procedures, such as rectal manometry and full-thickness rectal biopsy, were not performed since Hirschsprung disease was unlikely due to response, still partial and intermittent, to laxatives and the absence of typical barium enema images, showing reduced caliber of the rectum, followed by a transition zone to an enlarged-caliber sigmoid. He has no expressive language, independent sitting or rolling over, and eye contact has slightly improved. No abnormalities in coagulation, endocrine, liver, and renal function tests have been documented. He had a mild SARS-COV-2 infection, and no seizures were triggered by a mild increase in temperature. Due to severe constipation, pyridoxine (vitamin B6) and glucosamine supplementation could not be started. Bilateral ureteroceles and ureteropielectasis remain stable. The last EEG assessment revealed a very high-voltage, asynchronic, slow wave-spike pattern consistent with hypsarrhythmia, which clinically correlated with infantile spasms illustrated by head bobbing and nystagmoid eye movements; levetiracetam was withdrawn, and vigabatrin and topiramate were started, achieving good seizure control. According to the Nijmegen Pediatric CDG Rating Scale (NPCRS) (Achouitar et al., 2011), the patient’s score is 25, which scales him in the upper limit of the moderate category.

**FIGURE 2:**
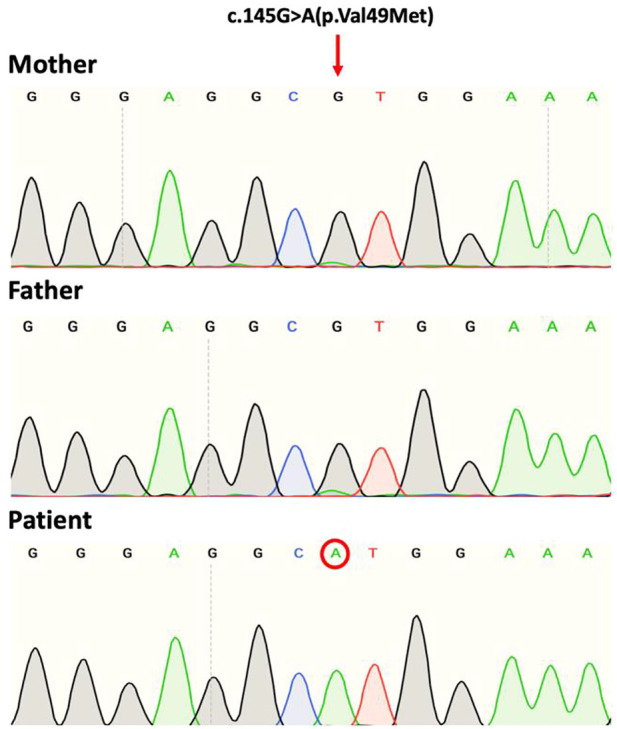
Sanger sequence chromatograms of gDNA showing the *PIGA* variant NM_002641.4(PIGA):c.145G>A (p.Val49Met). The patient shows the mutation at codon position 49 of exon 2. Both mother and father are non-carriers.

Exome sequencing of the child revealed the presence in exon 2 of the NM_002641.4(PIGA):c.145G>A (p.Val49Met) variant with genomic location X:15331786 (GRCh38). Sanger sequencing data identified that the variant is a *de novo* mutation in view that sequencing of parental gDNA showed that the mother was a non-carrier (Figure 2).

**FIGURE 3:**
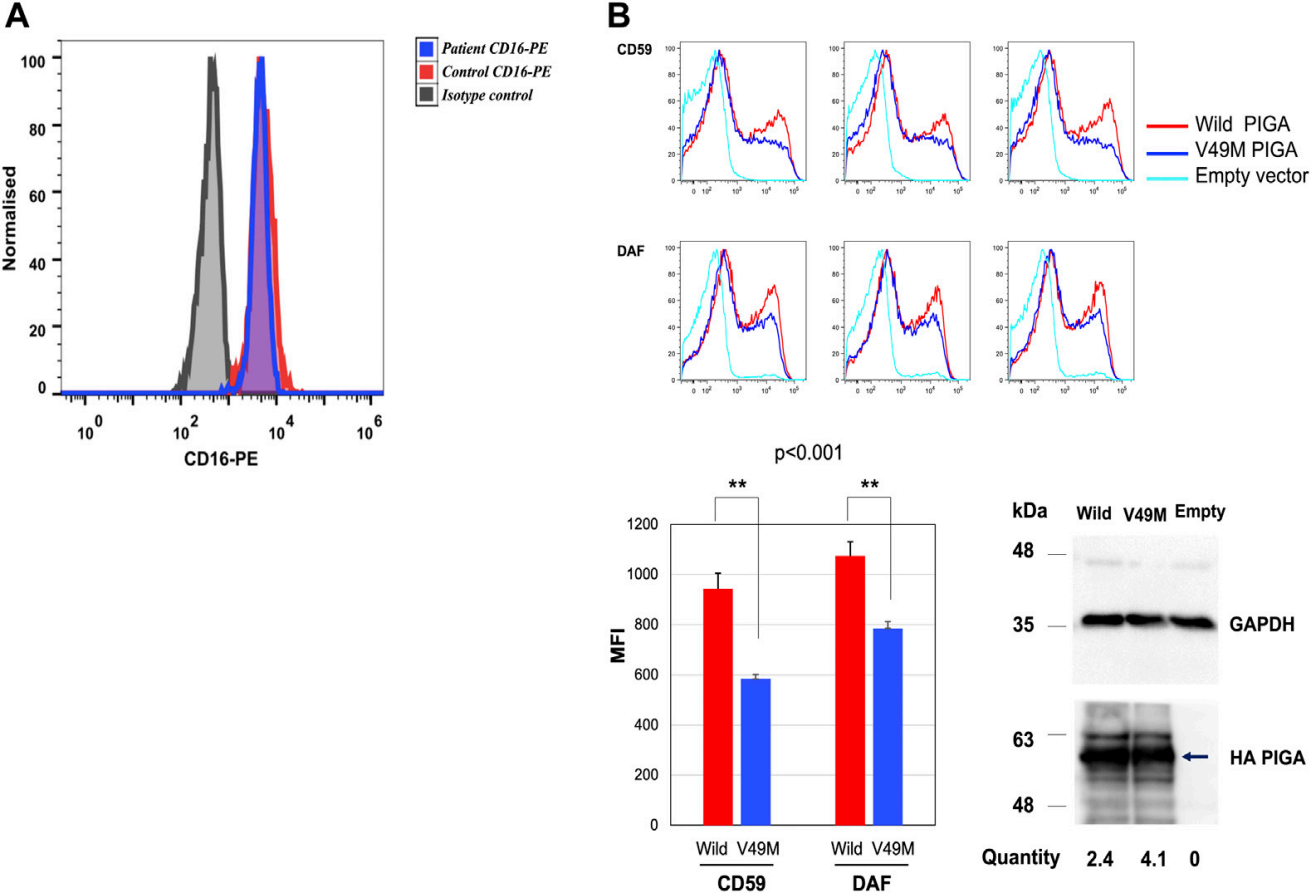
CD16 expression in granulocytes and rescue analysis in the PIGA-KO HEK293 cells. **(A)** Histogram depicting median fluorescence intensity (MFI) of CD16-PE in granulocytes from the patient (blue histogram) and healthy control (red histogram) (healthy control MFI = 5,130 *vs.* patient MFI = 4,450). **(B)** Rescue of CD59 and DAF expression on the PIGA-KO HEK293 cells using a weak TATA box only promoter (pTA). The histograms represent the MFI for the rescue of CD59 and DAF expression by the wild type and Val49Met (V49M) variant using the pTA promoter. Values for three independent experiments and their statistical significance were graphed. CD59: wild type MFI = 943 *vs.* variant MFI = 584. DAF: wild type MFI = 1,074 *vs.* variant MFI = 785. Representative Western blotting of the mutant (Val49Met) and wild type (Wild) *PIGA* protein expression showing that the *PIGA* mutant protein was more expressed than the wild-type protein. (normalized with the intensities of GAPDH, the loading control, and luciferase activities used for evaluating transfection efficiencies). *PIGA* protein was detected by anti-His mAb (arrow), and loading control was revealed with anti-GAPDH. Empty vector = vector without insert gene. Histograms in **(A)** y axis show cell counts; the x axis shows fluorescence intensity.

To determine the functional impact of the c.145G>A (p.Val49Met) variant, granulocyte expression of CD16 was determined by flow cytometry. CD16 is a GPI-anchored protein, considered a biomarker for IGDs (Bruneel et al., 2020). A 13% reduction in CD16 was observed in the patient compared to the healthy control (Figure 3A). This degree of reduction has been reported in other PIGA-CDG patients (Kim et al., 2016).

Additionally, a rescue analysis of DAF and CD59 expression was performed by flow cytometry in a HEK-293 PIGA KO model generated by the CRISPR/Cas9 system (Guerrero et al., 2021). DAF and CD59 are well-known GPI-anchored proteins used as biomarkers for IGDs and that are expressed in HEK-293 cells. CD16 is not expressed in this cell line.

HEK-293 PIGA KO cells were transfected with the wild type and the c.145G>A (p.Val49Met) variant, driven by the weak TATA box only promoter (pTA). Rescue of CD59 expression by the variant was found to be significantly deficient (62% of wild type; *p* < 0.001). Rescue of DAF expression was also found to be significantly deficient (73% of wild type; *p* < 0.001) (Figure 3B). Western blot from cell lysates showed similar expression of both wild type and mutant protein (Figure 3B).

## Discussion and conclusion

CDGs, including IGDs, are scarcely reported from Latin America. Less than 90 cases of germline PIGA have been reported worldwide, mostly in the severe phenotype spectrum. We report the case of a male child that presented with a predominantly neurological and gastrointestinal infection, including moderate and prolonged neonatal jaundice that was associated with Gilbert’s syndrome, caused by the TA6/TA7 *UGT1A1* (also named UGT1A1*28) polymorphism. The *UGT1A1* is related to autosomal recessive indirect hyperbilirubinemia syndromes. While some authors report the association of this polymorphism with mild-to-moderate neonatal hyperbilirubinemia (Roy-Chowdhury et al., 2002; Agrawal et al., 2009), others have failed to demonstrate its clinical significance alone on jaundice risk (Ülgenalp et al., 2003; Watchko and Lin, 2010). Nevertheless, the combination of the TA6/TA7 genotype with other icterogenic conditions, such as hemoglobinopathies, may increase the risk for hyperbilirubinemia (Watchko and Lin, 2010) and may play an additional role in the pathogenesis of hemolytic neonatal hyperbilirubinemia (Yang et al., 2021) by interfering with the bilirubin clearance pathway. Therefore, the occurrence of the variant described previously with the *PIGA* mutation may explain the prolonged neonatal hyperbilirubinemia, which is not expected nor observed in CDG patients (Marques-da-Silva et al., 2017; Bayat et al., 2020; Lipiński et al., 2021).

Exome sequencing of the patient’s gDNA revealed the presence of the variant NM_002641.4(PIGA):c.145G>A (p.Val49Met) ClinVar 623369 reported with conflicting interpretations of pathogenicity. The variant in this case is considered *de novo* as the mother was determined a non-carrier. The c.145G>A (p.Val49Met) variant has been reported in the literature in three male patients who acquired it through maternal inheritance (mothers were not reported to be affected). The first reported case was characterized by renal cysts with epileptic seizures and DD (Knaus et al., 2018); the second case showed profound DD, epileptic spasms, and focal seizures initiating at 2 months evolving to bilateral tonic-clonic seizures of intractable prognosis, with the patient dying at 12 years old suddenly and unexpectedly (Bayat et al., 2020); and the third case exhibited epileptic spasms and non-motor onset seizures with behavior arrest, myoclonic jerks, and apneas, developing pharmacoresistance with severe status epilepticus (Cabasson et al., 2020).

In contrast to the previous reports involving the 145G>A (p.Val49Met) variant, the patient exhibits a moderate neurological phenotype, perhaps related to the absence of documented brain malformations, refractory epilepsy, and multiorgan involvement. In a large number of PIGA-CDG patients, it was reported that only 10% of patients born alive belonged to the milder end of the spectrum, but only 3% of deceased patients belonged to this part of the spectrum (Bayat et al., 2021).

A distinctive feature in this case was the development of megacolon, possibly originated by a deficient development of the enteric nervous system. Reduction in the expression of GPI-linked proteins could explain gastrointestinal symptoms in PIGA-CDG, including megacolon. For example, the GPI-linked protein GFRα1 is a co-receptor of the glial cell line–derived neurotrophic factor (GDNF) that participates in a signaling system involved in the migration of neural crest cells into the gut, as well as regulation of neuronal survival and death (Cacalano et al., 1998; Uesaka et al., 2007).

The 145G>A (p.Val49Met) variant is located in the Rossmann A fold region, a hot spot for cluster pathogenic *PIGA* variants (Knaus et al., 2018; Bayat et al., 2020; Cabasson et al., 2020). The Val49Met change has been predicted to be probably damaging by both PolyPhen-2 (Adzhubei et al., 2013) and SIFT (Kumar et al., 2009) assessed with 29.4 as the score using Combined Annotation–Dependent Depletion (CADD) (Bayat et al., 2020) and a REVEL score of 0.702 (likely disease-causing) (Ioannidis et al., 2016). This variant is absent from the gnomAD database.

Although the Val49Met has the lowest Grantham-score of *PIGA* variants studied in a huge number of patients (Bayat et al., 2020), the Val residue is highly conserved in *PIGA* proteins from several organisms, which could explain the importance of this amino acid residue in *PIGA* function and the functional impact of the Val49M substitution, as was determined by the significantly reduced rescue of CD59 and DAF expression observed in the complementation assays using the HEK293 PIGA KO cells. Taking into consideration the experimental and theoretical data, we can conclude that the 145G>A (p.Val49Met) PIGA variant is pathogenic.

Unfortunately, there are no corrective treatments available for PIGA-CDG. Improvement has been observed in some patients upon initiation with ketogenic diets (Joshi et al., 2016), probably through stimulation of γ-amino butyric acid (GABA) production and reception, initiating an anti-epileptic effect (Daci et al., 2018), and because of their high content in omega-3 and omega-6 polyunsaturated fatty acids (PUFAs) that present modulatory effects on voltage-gated ion channels and thus a potential anti-epileptic effect (Taha et al., 2010). More recently, PIGA cell lines and mouse models have been used to evaluate compounds or drugs that were originally developed to address different diseases as an alternative to finding an effective treatment for PIGA-CDG (Olsen et al., 2017; Guerrero et al., 2021; Liu et al., 2021; Lukacs et al., 2021).

## Patient perspective

The patient is currently enrolled in the Natural History Study of the Frontiers in Congenital Disorders of Glycosylation Consortium of the National Institutes of Health, United States. The parents are working with other families affected by CDG to advance diagnosis and treatment for patients in Mexico and are actively seeking clinical trials for their son to participate in.

## Data Availability

The datasets for this article are not publicly available due to concerns regarding participant/patient anonymity. Requests to access the datasets should be directed to the corresponding author.
